# Metabolic profiling distinguishes three subtypes of Alzheimer's disease

**DOI:** 10.18632/aging.100801

**Published:** 2015-08-31

**Authors:** Dale E. Bredesen

**Affiliations:** ^1^ Mary S. Easton Center for Alzheimer's Disease Research, Department of Neurology, University of California, Los Angeles, CA 90095, USA; ^2^ Buck Institute for Research on Aging, Novato, CA 94945, USA

**Keywords:** inflammation, neurodegeneration, cognition, insulin resistance, biomarkers, dementia, dyscalculia

## Abstract

The cause of Alzheimer's disease is incompletely defined, and no truly effective therapy exists. However, multiple studies have implicated metabolic abnormalities such as insulin resistance, hormonal deficiencies, and hyperhomocysteinemia. Optimizing metabolic parameters in a comprehensive way has yielded cognitive improvement, both in symptomatic and asymptomatic individuals. Therefore, expanding the standard laboratory evaluation in patients with dementia may be revealing. Here I report that metabolic profiling reveals three Alzheimer's disease subtypes. The first is inflammatory, in which markers such as hs-CRP and globulin:albumin ratio are increased. The second type is non-inflammatory, in which these markers are not increased, but other metabolic abnormalities are present. The third type is a very distinctive clinical entity that affects relatively young individuals, extends beyond the typical Alzheimer's disease initial distribution to affect the cortex widely, is characterized by early non-amnestic features such as dyscalculia and aphasia, is often misdiagnosed or labeled atypical Alzheimer's disease, typically affects ApoE4-negative individuals, and is associated with striking zinc deficiency. Given the involvement of zinc in multiple Alzheimer's-related metabolic processes, such as insulin resistance, chronic inflammation, ADAM10 proteolytic activity, and hormonal signaling, this syndrome of Alzheimer's-plus with low zinc (APLZ) warrants further metabolic, genetic, and epigenetic characterization.

## INTRODUCTION

Alzheimer's disease represents a major healthcare problem, with over five million Americans estimated to suffer from this disease, and a recent study showing that AD has now become the third leading cause of death, trailing only cardiovascular disease and neoplasia [1]. The cause(s) of AD remain incompletely determined, and there is currently no truly effective treatment. However, accumulating data suggest important contributions from metabolic abnormalities such as insulin resistance, metabolic syndrome, chronic inflammation, hypovitaminosis D, hormonal deficiencies, and hyperhomocysteinemia, among others [2]. Despite this, most clinical evaluations of patients with cognitive decline do not include extensive metabolic or genomic evaluations. Furthermore, given the perceived poor prognosis for AD, in patients with evidence of amyloid-β accumulation by amyloid PET imaging or, indirectly, by cerebrospinal fluid profile, there has been little incentive to perform extensive evaluations of hormonal status, nutritional status, toxicity status, metal status, gastrointestinal permeability, or other laboratory evaluations perceived by healthcare systems as “non-standard.” However, studies such as the recent FINGER study [3] suggest that metabolic factors may play important roles in the neurodegenerative process, at least early in the pathogenetic process. Recent results from the evaluation of neural exosomes and nanosomes support the notion that metabolic abnormalities are present in patients with cognitive decline, often years prior to diagnosis of AD [4]. Therefore, it may be productive, both from the standpoint of identifying novel biomarkers and from the standpoint of identifying treatable metabolic abnormalities, to perform metabolic profiling of patients with cognitive decline and those at risk for such decline.

I recently described a protocol for metabolic enhancement in neurodegeneration [5]. Evaluating these same metabolic parameters in patients with cognitive decline revealed three subtypes of Alzheimer's disease, which are described in greater detail below. Such metabolic subtyping may provide novel insights into the pathogenesis in specific patients, as well as suggesting therapeutic approaches that may be effective only in specific subgroups of patients.

## RESULTS

### Case studies

#### Subtype 1: Inflammatory

It has been well documented via numerous methods and observations that inflammation plays an important role in AD pathogenesis. Among the many findings implicating inflammation in AD mechanisms: the presence of pro-inflammatory cytokines, chemokines, acute-phase reactants, and other mediators of inflammation in AD brains [6, 7]; the mutual antagonism between NFκB (nuclear factor κ-light-chain enhancer of activated B cells) and the sirtuin SirT1 [8] is altered in favor of inflammation, with reduced SirT1, in the brains of patients with AD [9]; genomic studies implicate multiple inflammation-associated genes in AD [10]; and phagocytosis of amyloid-β peptide is reduced by inflammation in patients with AD [11]. Moreover, a recent integrative analysis of AD susceptibility factors in the context of the signaling networks and molecular mechanisms mediating Alzheimer's disease pathophysiology implicated persistent wound-like microenvironments/pockets as playing a potentially causative role in AD etiology [12]. However, unlike other neuro-inflammatory diseases such as multiple sclerosis and encephalitides, the inflammation in AD involves primarily the innate immune system [6]. The pathology of AD includes inflammatory microglia and activated astroglia, and many but not all patients with AD show evidence of systemic inflammation, as well:

A 65-year-old man presented with a four-year history of progressive memory loss. He had had a superior memory for his entire life, and in fact had been known for his prodigious memory, but in his early 60s he began to have “senior moments” and to feel tentative about his driving directions. Both parents had died with dementia. Quantitative neuropsychological testing suggested a diagnosis of mild cognitive impairment. He was found to be heterozygous for the ε4 allele of Apolipoprotein E (3/4) and for MTHFR (methylene tetrahydrofolate reductase) A1298C. Magnetic resonance imaging was read as “normal, with hippocampal volume at 17^th^ percentile for age.” Fluorodeoxyglucose PET scan was abnormal, revealing a pattern typical of AD, with temporal and parietal reductions in glucose utilization. Amyloid PET scan was positive.

His high-sensitivity C-reactive protein was 9.9mg/l, albumin:globulin ratio 1.6, fasting insulin 32uIU/ml, fasting serum glucose 96mg/dl, homocysteine 15.1uM, 25-hydroxycholecalciferol 21ng/ml, testosterone 264ng/dl, pregnenolone <5ng/dl, TSH 2.21mIU/l, free T3 2.4pg/ml, free T4 0.8pg/ml, vitamin B12 328pg/ml, serum zinc 98mcg/dl, serum ceruloplasmin 26mg/dl, and serum mercury 19ng/ml. BMI (body mass index) was 25.

#### Subtype 2: Non-inflammatory

In addition to inflammatory mechanisms in AD, numerous alternative associations have been described, such as insulin resistance, hypovitaminosis D, hyper-homocysteinemia, and hormonal loss associated with early oophorectomy [2, 13]. The extent to which each of these, beyond being a risk factor, contributes directly to AD pathogenesis is incompletely defined. Nonetheless, for each of these, as well as other metabolic mediators, there are theoretical mechanisms to support contributions to AD pathogenesis. Homocysteine, for example, has been shown to exert multiple effects that may contribute to cognitive decline, such as increasing tau phosphorylation via a post-translational modification-mediated reduction in protein phosphatase 2A (PP2A) function [14], glutamate receptor dysfunction, neuronal apoptosis induction, endoplasmic reticulum stress, DNA methylation, mitochondrial dysfunction, vascular damage, and oxidative stress [15].

A 75-year-old woman had progressive memory loss over one year. She had been otherwise healthy. Her mother had developed dementia in her eighth decade, as well. Her MoCA was 17/30. Her hs-CRP was 0.7mg/l, hemoglobin A1c 5.4%, 25-hydroxycholecalciferol 34ng/ml, homocysteine 24.1uM, MTHFR C677T heterozygous, vitamin B12 338pg/ml, MCV 102fl, pregnenolone 40ng/dl, TSH 3.5mIU/l, free T3 2.7pg/ml, free T4 1.0pg/ml, AM cortisol 21mcg/dl, and serum zinc 82mcg/dl.

#### Subtype 3: Cortical

Alzheimer's disease presenting with non-amnestic features such as aphasia, partial Gerstmann's syndrome, alexia, visual agnosia, or apraxias has been well described [16], but metabolic abnormalities distinguishing these atypical presentations have not been reported. These patients represent a group that is distinct from the more typical amnestic presentation in several respects: (1) early symptom onset, typically in the 5^th^-7^th^ decades; (2) lack of family history; (3) ApoE4-negative in the majority; (4) MRI showing general cortical atrophy (and in some cases, cerebellar atrophy, as well) rather than hippocampal atrophy out of proportion to the rest of the cerebrum; (5) FDG-PET may show reductions in glucose utilization beyond the typical temporal-parietal distribution. A summary of six patients with this presentation is shown in Table 1.

**Table 1:** Patients with the third subtype of Alzheimer's disease described in the text, Alzheimer's-plus with low zinc

Patient	Age at onset	Initial Symptoms	ApoE4?	Zinc	Other
1M	65	Visual agnosia	− (3/3)	56	MRI:general atrophy, mild FLAIR
2M	59	Dyscalculia, aphasia	− (2/3)	59	MRI:general atrophy, mild FLAIR; FDG PET: frontal, temporal, parietal abnl.
3F	50	Dyscalculia	− (3/3)	56	MRI:general atrophy, mild FLAIR; CSF +
4F	64	Dyscalculia, prosopagnosia, word finding	Declined	59	Cu:Zn=3:1
5M	55	Dyscalculia	− (3/3)	ND	MRI:general atrophy, CSF +
6F	57	Dyscalculia	+ (3/4)	70	MRI:general atrophy, mild FLAIR; amyloid PET +

A 52-year-old scientist presented with a two-year history of cognitive decline, which had started with difficulty with numbers: she was unable to tip, unable to pay bills, and then after several months she asked for help to write a grant proposal. She declined rapidly and developed a simple, childlike affect.

Despite this, she was able to learn and remember the names of all 28 children on the playground at her son's school. Family history was negative. Her MoCA score was 19. Her MRI showed “global cerebral volume loss, advanced for age.” There were several areas of FLAIR (fluid-attenuated inversion recovery) hyperintensity in the subcortical and periventricular white matter. In addition, there was atrophy of the superior cerebellar vermis, and to a lesser degree the cerebellar hemispheres. CSF was markedly abnormal, with reduced Aβ42 of 294pg/ml and increased p-tau of 133pg/ml, compatible with a diagnosis of Alzheimer's disease.

BMI was 24.9. She was ApoE 3/3, klotho variant negative (SNP Rs9536314), hs-CRP was 1.4mg/l, albumin:globulin ratio 1.57, IL-6 1.4pg/dl, hemoglobin A1c 5.3%, fasting insulin 4.5mIU/l, TSH 2.14mIU/l, free T3 4.2pg/ml, reverse T3 11ng/dl, free T4 1.0pg/ml, progesterone < 0.21ng/ml, estradiol 3pg/ml, 17-hydroxypregnenolone 14ng/dl, AM cortisol 9mcg/dl, 25-hydroxycholecalciferol 22ng/ml, total cholesterol 264mg/dl, HDL-cholesterol 67mg/dl, LDL-cholesterol 167mg/dl, triglycerides 61mg/dl, cholesterol:HDL ratio 3.7, serum copper 101mcg/dl, serum zinc 56mcg/dl, and Cu:Zn ratio 1.8.

A 59-year-old man began to have word-finding difficulties, followed by difficulties with arithmetic. He had been a type A personality with a high-powered position, whose symptoms had begun after two years of the most stressful time of his career, and he became very passive and timid. Neuropsychological testing showed profound impairment in semantic fluency, executive functioning, attention, overall mental status, processing, and visual memory. Fluorodeoxyglucose PET scanning showed reduced metabolism in temporal and parietal lobes, L>R precuneus, and left frontal lobe.

His BMI was 24.9, ApoE genotype 2/3, hs-CRP 0.5mg/l, albumin 4.5g/dl, globulin 2.4g/dl, albumin:globulin ratio 1.9, AM cortisol 15.8mcg/dl, total cholesterol 235mg/dl, HDL-cholesterol 70mg/dl, LDL-cholesterol 150mg/dl, triglycerides 75mg/dl, cholesterol:HDL ratio 3.4, DHEA-S 130mcg/dl, progesterone 0.4ng/ml, fasting insulin 6mIU/l, 25-hydroxycholecalciferol 44.5ng/ml, alpha-tocopherol 22.5mg/l, beta-gamma-tocopherol 0.5mg/l, TSH 2.98mIU/l, free T3 2.7ng/ml, free T4 1.2ng/dl, reverse T3 21ng/dl, pregnenolone <5ng/dl, homocysteine 7.3umol/l, folate 16.6ng/ml, RBC Mg 5.5mg/dl, serum iron 135mcg/dl, serum copper 97mcg/dl, serum zinc 59mcg/dl, Cu:Zn ratio 1.6, blood arsenic/lead/mercury all <2mcg/l, TNF 1.2pg/ml, and IL-6 1.7pg/ml.

Sleep study showed mild obstructive sleep apnea, with apnea/hypopnea index of 7 events per hour. No REM behavioral disturbance was noted.

## DISCUSSION

The classification of Alzheimer's disease into subtypes may be useful for therapeutic studies. Since increasing evidence supports an important role for metabolic abnormalities such as insulin resistance in Alzheimer's disease pathophysiology, it is of interest to determine whether metabolic profiling may be useful clinically, both in classification and, ultimately, in therapeutic trials.

Here it is shown that clinical metabolic testing reveals three readily distinguishable subtypes of Alzheimer's disease. The first type, inflammatory, is associated with markers of systemic inflammation, such as high hs-CRP, low albumin:globulin ratio, and high interleukin-6. Additional metabolic abnormalities may also be present, such as insulin resistance, metabolic syndrome, hyperhomocysteinemia, hypovitaminosis D, hypo-thyroidism, and hypercortisolemia. ApoE4, which exerts pro-inflammatory effects, is associated with this subtype of Alzheimer's disease. The presentation is typically amnestic, and imaging studies are compatible with such a presentation, showing hippocampal atrophy in the absence of widespread cerebral atrophy.

The second type, non-inflammatory, is associated with other metabolic abnormalities such as insulin resistance, hypovitaminosis D, hyperhomocysteinemia, and reductions in hormonal support. Indicators of systemic inflammation, such as high hs-CRP, reduced albumin:globulin ratio, and high interleukin-6, are by definition absent, but of course this does not exclude the possibility of intra-CNS inflammation. The patients with this second subtype of AD tend to be slightly older than those in the first, often in the eighth instead of seventh decade. Interestingly, Fiala's studies have shown that both inflammatory and non-inflammatory subtypes display phagocytic defects for amyloid-β in their peripheral blood mononuclear cells [17]. This second subtype is also associated with ApoE4, and, as for the inflammatory subtype, tends to present with an amnestic syndrome and FDG-PET showing temporal-parietal reductions in glucose utilization.

In contrast to the profiles for the first two subtypes, the profile of the third subtype argues that it is a fundamentally different disease process than the first two: instead of losing the ability to form new memories as the initial presentation, in this subtype there is an initial loss of long-term memory maintenance, resulting in problems such as dyscalculia and aphasia, with an initial retention of new memory formation and retrieval. A recurring feature was the patients' ability to describe recent events in detail, but frequent inability to keep their trains of thought. In some cases, the presentation was associated with a history of depression. However, none had the visual hallucinations, delusions, REM behavioral disturbance, or autonomic disorders that would suggest Lewy body dementia. Some of the patients were also noted to become passive, simple-minded, or childlike, often in marked contrast to their earlier, highly-accomplished, hard-driving personalities. There was widespread cerebral atrophy (sometimes along with cerebellar atrophy), and early symptom onset in the fifth, sixth, or early seventh decades, but no ApoE4-related over-representation and typically no family history of Alzheimer's disease. Surprisingly, all had very low serum zinc, typically 50-60mcg/dl. It should be noted that serum zinc is a relatively insensitive test for zinc deficiency, so that low serum zinc is strongly suggestive of relatively severe zinc deficiency, but a normal serum zinc does not necessarily exclude some degree of zinc deficiency.

Zinc is the second most abundant trace metal in the human body, following only iron. Over 300 enzymes utilize zinc as a co-factor, either for catalysis (in which it functions as a Lewis acid) or structure, and it typically sits in a distorted tetrahedral structure, coordinated with three or four protein side chains and interacting directly with a cysteine sulfur and/or histidine nitrogen and/or glutamate or aspartate oxygen [18]. Zinc deficiency, which is common in aging individuals, affects many functions that are directly or indirectly related to cognitive performance and Alzheimer's disease. For example, zinc deficiency induces insulin resistance, a known risk factor for AD. Zinc deficiency also increases inflammation, and reduces the finely tuned nature of the immune response, resulting in a reduced specific response (and thus loss of resistance to infectious agents) and a greater autoimmune response. Zinc is also involved in wound healing, DNA repair, and oxidative damage. Zinc deficiency is associated with increased aging, increased susceptibility to toxins, increased susceptibility to infections, increased production of reactive oxygen species, reduced hormonal function, reduced adrenal support, increased susceptibility to copper toxicity, and gastrointestinal hyperpermeability. Furthermore, zinc treatment has been shown to mitigate cognitive decline [19]. Therefore, zinc deficiency is a concern as a potential contributor to cognitive decline.

Zinc deficiency is relatively common, and may be associated with poor absorption due to reduced gastric acidity (e.g., due to H. pylori and/or the use of proton pump inhibitors), a zinc-deficient diet (e.g., due to vegetarianism without supplementation), adrenal stress, diabetes, alcohol use, toxic exposure, intestinal parasites, or aging. As noted above, serum zinc represents a relatively insensitive test for zinc; RBC zinc provides a more accurate assessment. Thus by the time that serum zinc is low, the body's zinc deficiency is likely to be quite severe.

The cause(s) of zinc deficiency in the six patients reported here is unknown, as is the potential relationship of the zinc deficiency to the distinctive neurodegenerative process. Given the relationship between zinc deficiency and susceptibility to toxins and infectious agents, historical data were obtained from all patients on potential toxic exposure. Whether or not causally related, all had histories of toxic exposures: one had grown up in Tom's River, New Jersey, a well documented area of extreme chemical toxicity; another had a sibling with childhood leukemia (which may suggest exposure to chemical toxins) and himself had worked for a chemical company for years, describing the difficulty with dealing with the severe chemical odors. Two others had lived in a home heavily contaminated with molds for years, another had worked with sewage for many years, and another had had unusually extensive dental amalgam work over the years.

In summary, metabolic profiling of patients with cognitive decline, as described previously [5], reveals three readily distinguishable subtypes of Alzheimer's disease: inflammatory, non-inflammatory, and cortical. The distinctive features, presentation, lack of association with ApoE4, and marked hypozincemia, together suggest that the cortical subtype of Alzheimer's disease is a fundamentally different disease than the other two subtypes. This subtype deserves further genetic, epigenetic, and metabolomic studies.
